# Coping Strategies and Symptoms of Mental Health Disorders Among People with HIV Initiating HIV Care in Cameroon

**DOI:** 10.1007/s10461-022-03963-4

**Published:** 2023-01-06

**Authors:** Angela M. Parcesepe, Lindsey M. Filiatreau, Amanda Gomez, Peter Vanes Ebasone, Anastase Dzudie, Brian W. Pence, Milton Wainberg, Marcel Yotebieng, Kathryn Anastos, Eric Pefura-Yone, Denis Nsame, Rogers Ajeh, Denis Nash

**Affiliations:** 1 Department of Maternal and Child Health, Gillings School of Global Public Health, University of North Carolina at Chapel Hill, Chapel Hill, NC USA; 2 Carolina Population Center, University of North Carolina at Chapel Hill, Chapel Hill, NC USA; 3 Department of Psychiatry, School of Medicine, Washington University in St. Louis, St. Louis, MO USA; 4 Clinical Research Education Networking and Consultancy, Yaoundé, Cameroon; 5 Department of Epidemiology, Gillings School of Global Public Health, University of North Carolina at Chapel Hill, Chapel Hill, NC USA; 6 Department of Psychiatry, Columbia University and New York State Psychiatric Institute, New York, NY USA; 7 Department of Medicine, Albert Einstein College of Medicine, Bronx, NY USA; 8 Departments of Medicine and Epidemiology & Population Health, Albert Einstein College of Medicine, Bronx, NY USA; 9 Jamot Hospital, Yaoundé, Cameroon; 10 Bamenda Regional Hospital, Bamenda, Cameroon; 11 Institute for Implementation Science in Population Health, City University of New York, New York, NY USA

**Keywords:** Coping, Depression, Anxiety, PTSD, HIV, Cameroon

## Abstract

Little is known about the coping strategies used among people with HIV (PWH), especially in sub-Saharan Africa, and the extent to which adaptive or maladaptive coping strategies are associated with symptoms of mental health disorders. We interviewed 426 PWH initiating HIV care in Cameroon and reported the prevalence of adaptive and maladaptive coping strategies, overall and by presence of symptoms of depression, anxiety, and PTSD. Log binominal regression was used to estimate the association between each type of coping strategy (adaptive or maladaptive) and symptoms of each mental health disorder, separately. Adaptive and maladaptive coping strategies were commonly reported among PWH enrolling in HIV care in Cameroon. Across all mental health disorders assessed, greater maladaptive coping was associated with higher prevalence of depression, anxiety, and PTSD. Adaptive coping was not associated with symptoms of any of the mental health disorders assessed in bivariate or multivariable models. Our study found that PWH endorsed a range of concurrent adaptive and maladaptive coping strategies. Future efforts should explore the extent to which coping strategies change throughout the HIV care continuum. Interventions to reduce maladaptive coping have the potential to improve the mental health of PWH in Cameroon.

## Introduction

Advances in HIV treatment have increased life expectancy and improved quality of life among people with HIV (PWH) [1–3]. As a result of increased access to and efficacy of antiretroviral therapy (ART), HIV can now be managed as a chronic disease. Despite significant advances in HIV treatment, PWH continue to experience numerous psychosocial stressors including HIV-related stigma, interpersonal conflict and violence related to HIV disclosure, HIV- and non-HIV-related grief and loss, and HIV- and non-HIV-related material hardship [4–6]. This is particularly relevant among PWH in sub-Saharan Africa (SSA) where the majority of PWH reside and where the burden of material hardship, interpersonal violence, and HIV-related stigma has been well-documented [7–9].

PWH are also at increased risk of mental health disorders across global settings [10, 11]. It has been estimated that approximately half of PWH screen positive for one or more mental or substance use disorders. Psychosocial stressors have been associated with increased prevalence of mental and substance use disorders among PWH [7, 12, 13]. Psychosocial stressors and mental and substance use disorders have been consistently associated with poor HIV treatment outcomes, including engagement into HIV care with advanced HIV, delayed ART initiation, poor ART adherence, and virologic failure [14–17].

The chronic nature of HIV infection and the high prevalence of psychosocial stressors and mental health disorders among PWH call for better understanding of the ways in which PWH cope with stressful situations. Coping has been defined as the cognitive and behavioral strategies used to manage situations that tax or exceed the resources of an individual [18]. A wide range of coping strategies exist and vary across cultures [19, 20]. Coping strategies have been commonly categorized as adaptive or maladaptive [21]. Adaptive coping strategies include those that foster active engagement with psychosocial stressors in ways that may impact positive change and typically include planning, acceptance, positive reframing, and the use of emotional support, among others. Adaptive strategies have been associated with improved mental health and reduced stress [22, 23]. Among PWH, adaptive coping strategies have also been associated with better HIV treatment outcomes, including greater ART adherence and health-related quality of life [24–26]. Maladaptive coping strategies include those that foster avoidance or disengagement from psychosocial stressors and include strategies such as distraction, disengagement, denial, and substance use, among others. Maladaptive strategies have been associated with increased stress and worse mental health [27, 28]. Among PWH, maladaptive strategies have been associated with suboptimal HIV treatment outcomes, including delayed ART initiation, poor ART adherence, and lower likelihood of viral suppression [29–31].

Little is known about the coping strategies used among PWH, especially in SSA where PWH are at substantial risk of economic and psychosocial stressors, and the extent to which coping strategies are associated with symptoms of mental health disorders among PWH in SSA. A meta-analysis of coping strategies among PWH found that maladaptive coping strategies of disengagement and substance use were associated with worse mental and physical health while adaptive coping strategies of active coping and positive reframing were associated with better mental and physical health [32]. However, this meta-analysis only included studies from high-income countries. Among studies that have examined coping among PWH in SSA, findings are equivocal. Research with PWH in Botswana and pregnant women with HIV in South Africa found that adaptive coping strategies were negatively associated with depressive symptoms and maladaptive coping strategies were positively associated with depressive symptoms [33, 34]. However, a study among women with HIV in Uganda found that adaptive coping strategies were positively associated with depression and anxiety symptoms [35].

Coping strategies are potentially modifiable mechanisms that may improve the mental health and well-being of PWH. Given the prevalence of mental health disorders among PWH in SSA and the well-established mental health treatment gap in the region, greater understanding of coping among PWH in SSA and the relationship between coping and mental health can inform the development and implementation of coping-related interventions to improve the mental health and quality of life of PWH in SSA [36, 37]. This research sought to: assess the construct validity of the Brief Coping Orientation to Problems Experienced Scale (Brief COPE), describe the prevalence of unique coping strategies, and examine the relationship between coping strategies and symptoms of depression, anxiety, and post-traumatic stress disorder (PTSD) in a population of PWH entering HIV care in Cameroon.

## Methods

### Data Collection

This study has been previously described [15]. Briefly, interviewer-administered surveys were conducted with 426 individuals initiating HIV care at three HIV clinics in Cameroon between June 2019 and March 2020. Surveys were conducted in English or French, based on the participant’s preference. Individuals were eligible to participate if they were 21 years or older and enrolling in HIV care at one of three HIV clinics. Data collection included questions on mental health, substance use, coping mechanisms, and sociodemographic characteristics. Interviewers received training to minimize influencing participants’ responses. This study was approved by the Institutional Review Board at the University of North Carolina at Chapel Hill and the National Ethical Committee of Research for Human Health in Cameroon.

### Measures

#### Coping Strategies

Coping strategies were assessed using the Brief Coping Orientation to Problems Experienced Scale (Brief COPE) [38]. The Brief COPE consists of 28 questions about 14 different coping strategies including: distraction, active coping, denial, substance use, use of emotional support, use of instrumental support, behavioral disengagement, venting, positive reframing, planning, humor, acceptance, religion, and self-blame. Participants were asked if they engaged in each coping strategy not at all, a little, a medium amount, or a lot. Based on the results of an exploratory factor analysis, the Brief COPE was divided into two factors: adaptive coping (14 items) and maladaptive coping (8 items). Adaptive coping was comprised of the following coping strategies: active coping, use of emotional support, use of instrumental support, positive reframing, religion, planning, acceptance, and humor. Maladaptive coping was comprised of the following strategies: self-blame, venting, behavioral disengagement, and denial. A sum score was calculated separately for the adaptive and maladaptive subscales and categorized into tertiles based on the distribution in the population.

#### Depressive Symptoms

Depressive symptoms were assessed with the Patient Health Questionnaire-9 (PHQ-9) [39]. The PHQ-9 assesses the presence of depressive symptoms in the last two weeks. Scores of 10 or greater were categorized as moderate to severe depressive symptoms [39]. The PHQ-9 has been validated in French and with PWH in SSA [40–43].

#### Anxiety Symptoms

Anxiety symptoms were assessed with the General Anxiety Disorder-7 (GAD-7) [44]. The GAD-7 assesses the presence of anxiety symptoms in the past two weeks. Scores of 10 or greater were categorized as moderate or severe anxiety symptoms. The GAD-7 has been validated in French and among a primary care population with a high prevalence of HIV in SSA [45–48].

#### Post-traumatic Stress Disorder Symptoms

Post-traumatic stress disorder (PTSD) symptoms were assessed with the PTSD Checklist for DSM-5 (PCL-5) [49]. The PCL-5 assesses the presence of PTSD symptoms in the past month. Scores of 31 or greater were categorized as symptoms of probable PTSD [50]. The PCL-5 has been validated in French and among a primary care population with a high prevalence of HIV in Zimbabwe [51, 52].

#### Sociodemographic Characteristics

Sociodemographic characteristics explored included age, gender, education, relationship status, employment, number of children, and household hunger.

### Missing Data

A small number of individuals were missing data on individual mental health scale items (n = 13 for PCL-5; n = 6 for PHQ-9; n = 12 for GAD-7). For individuals missing data on less than 10% of items for any given scale (n = 13 for PCL-5; n = 6 for PHQ-9; n = 10 for GAD-7), the mean of the individual’s non-missing scale responses was imputed for the missing items [53].

### Statistical Analyses

To explore the construct validity of the Brief COPE, we conducted an exploratory factor analysis using the principle components method and a varimax rotation. In determining the number of factors to retain, scree plots, Eigenvalue loadings, and the overall interpretability of the resulting factors were considered. Items with communality estimates below 0.20 upon initial inspection were dropped [54]. Subsequent to this, items with factor loadings less than 0.40 or cross-loadings greater than 70% were dropped.

We used counts, proportions, medians, and interquartile ranges to describe the study population and the prevalence of coping strategies among study participants overall and by presence of mental health symptoms. Log binominal regression was used to estimate the association between each type of coping strategy (adaptive or maladaptive) and symptoms of each mental health disorder, separately. Adjusted analyses controlled for age, gender, and clinic, the a priori covariates of interest. Clinic was included as a fixed effect variable given the small number of clinics in the study.

## Results

Most respondents were female (58.7%), 21–39 years of age (58.5%) and in a romantic relationship (58.5%) (Table 1). The exploratory factor analysis of the original 28-item Brief COPE identified a two-factor structure that accounted for 81% of the total variance. Both items from the active coping, use of emotional support, use of instrumental support, positive reframing, religion, and planning subscales and one item each from the acceptance and humor subscales loaded on Factor 1 which was named ‘adaptive coping strategies.’ Both items from the self-blame, venting, disengagement, and denial subscales loaded on Factor 2 which was named ‘maladaptive coping.’ Both items from the distraction and substance use subscales and one item each from the humor and acceptance subscales did not load onto the two-factor structure. Therefore, the exploratory factor analysis identified 22 items from the original Brief COPE that loaded onto two subscales of adaptive and maladaptive coping.

**Table 1:** Demographic characteristics of 426 people initiating HIV care in Cameroon, overall and stratified by coping strategies

	Total	Maladaptive coping			χ^2^, p-value	Adaptive coping			χ^2^, p-value
	(N = 426) N (col %)	Low (N = 134) N (row %)	Medium (N = 155) N (row %)	High (N = 137) N (row %)		Low (N = 144) N (row %)	Medium (N = 145) N (row %)	High (N = 137) N (row %)	
Age									
21–39	249 (58.5)	70 (28.1)	85 (34.1)	94 (37.8)	8.8, 0.01	80 (32.1)	89 (35.7)	80 (32.1)	1.0, 0.60
40 +	177 (41.5)	64 (36.2)	70 (39.5)	43 (24.3)		64 (36.2)	56 (31.6)	57 (32.2)	
Gender									
Female	250 (58.7)	70 (28.0)	103 (41.2)	77 (30.8)	6.5, 0.04	81 (32.4)	87 (34.8)	82 (32.8)	0.5, 0.77
Male	176 (41.3)	64 (36.4)	52 (29.5)	60 (34.1)		63 (35.8)	58 (33.0)	55 (31.3)	
Highest level of education									
None	31 (7.3)	5 (16.1)	14 (45.2)	12 (38.7)	12.1, 0.02	13 (41.9)	6 (19.4)	12 (38.7)	4.7, 0.32
Primary	218 (51.2)	58 (26.6)	83 (38.1)	77 (35.3)		72 (33.0)	72 (33.0)	74 (33.9)	
≥ Secondary	177 (41.5)	71 (40.1)	58 (32.8)	48 (27.1)		59 (33.3)	67 (37.9)	51 (28.8)	
Relationship									
Single	177 (41.5)	40 (22.6)	65 (36.7)	72 (40.7)	14.4, < 0.001	53 (29.9)	55 (31.1)	69 (39.0)	6.5, 0.04
Partnered	249 (58.5)	94 (37.8)	90 (36.1)	65 (26.1)		91 (36.6)	90 (36.1)	68 (27.3)	
Away from home > 1 Month in Last Year									
Yes	164 (38.5)	53 (32.3)	51 (31.1)	60 (36.6)	3.7, 0.15	60 (36.6)	53 (32.3)	51 (31.1)	0.9, 0.63
No	262 (61.5)	81 (30.9)	104 (39.7)	77 (29.4)		84 (32.1)	92 (35.1)	86 (32.8)	
Number of children									
0	79 (18.6)	25 (31.6)	27 (34.2)	27 (34.2)	0.3, 0.85	25 (31.7)	25 (31.7)	29 (36.7)	0.9, 0.65
1 +	345 (81.4)	109 (31.6)	128 (37.1)	108 (31.3)		119 (34.5)	118 (34.2)	108 (31.3)	
Missing	2	0	0	2		0	2	0	
Employment									
Employed	275 (64.6)	94 (34.2)	92 (33.5)	89 (32.4)	3.7, 0.16	93 (33.8)	98 (35.6)	84 (30.5)	1.2, 0.55
Unemployed	151 (35.4)	40 (26.5)	63 (41.7)	48 (31.8)		51 (33.8)	47 (31.1)	53 (35.1)	
Household hunger									
No/little	304 (71.9)	109 (35.9)	109 (35.9)	86 (28.3)	11.8, < 0.01	105 (34.5)	104 (34.2)	95 (31.3)	0.7, 0.69
Moderate/severe	119 (28.1)	24 (20.2)	45 (37.8)	50 (42.0)		37 (31.1)	40 (33.6)	42 (35.3)	
Missing	3	1	1	1		2	1	0	

Younger age (χ^2^ = 8.79, p = 0.01), being female (χ^2^ = 6.50, p = 0.04), lower levels of formal education (χ^2^ = 12.12, p = 0.02), not being in a romantic relationship (χ^2^ = 14.39, p < 0.01), and experiencing moderate or severe household hunger (χ^2^ = 11.80, p < 0.01) were associated with greater endorsement of maladaptive coping strategies (Table 1). In contrast, not being in a romantic relationship (χ^2^ = 6.50, p = 0.04) was the only sociodemographic characteristic assessed that was associated with greater endorsement of adaptive coping strategies (Table 1).

Religious coping was the most commonly reported coping strategy: 70.4% of participants reported that they prayed or meditated a medium amount or a lot and 68.8% that they tried to find comfort in their religion or spiritual beliefs a medium amount or a lot (Table 2). The least frequently used coping strategy was substance use with 13.8% reporting that they used alcohol or drugs to feel better a medium amount or a lot and 11.3% reporting that they used alcohol or drugs to get through difficult situations a medium amount or a lot.

**Table 2:** Frequency of coping strategy use among 426 people initiating HIV care in Cameroon

	Medium amount/a lot N (%)
Adaptive coping strategies	
Concentrating your efforts on doing something about the situation you’re in (active coping)	226 (53.1)
Taking action to try to make the situation better (active coping)	260 (61.0)
Getting emotional support from others (use of emotional support)	197 (46.2)
Getting comfort and understanding from someone (use of emotional support)	268 (62.9)
Trying to get advice or help from other people about what to do (use of informational support)	245 (57.5)
Getting help and advice from others (use of informational support)	249 (58.5)
Trying to see it in a different light, to make it seem more positive (reframing)	228 (53.5)
Looking for something good in what is happening (reframing)	249 (58.5)
Praying or meditating (religion)	300 (70.4)
Trying to find comfort in your religion or spiritual beliefs (religion)	293 (68.8)
Trying to come up with a strategy about what to do (planning)	249 (58.5)
Thinking hard about what steps to take (planning)	247 (58.0)
Accepting the reality of the fact that it has happened (acceptance)	254 (59.6)
Making fun of the situation (humor)	104 (24.4)
Maladaptive coping strategies	
Criticizing yourself (self-blame)	144 (33.8)
Blaming yourself for things that happened (self-blame)	171 (40.1)
Expressing your negative feelings (venting)	120 (28.2)
Saying things to let your unpleasant feelings escape (venting)	113 (26.5)
Giving up the attempt to cope (disengagement)	62 (14.6)
Giving up trying to deal with it (disengagement)	87 (20.4)
Refusing to believe that it has happened (denial)	63 (14.8)
Saying to yourself “this isn’t real” (denial)	93 (21.8)
Other	
Turning to work or other activities to take your mind off things (distraction)	196 (46.0)
Doing something to think about it less, such as going to movies, watching TV, reading, daydreaming, sleeping, or shopping (distraction)	277 (65.0)
Using alcohol or other drugs to feel better (substance use)	59 (13.8)
Using alcohol or other drugs to help you get through it (substance use)	48 (11.3)
Making jokes about it (humor)	111 (26.1)
Learning to live with it (acceptance)	216 (50.7)

Participants reported a variety of concurrent coping mechanisms (Table 3). Overall, 16.4% of participants reported low levels of both adaptive and maladaptive coping and 15.7% reported high levels of adaptive and maladaptive coping. On the contrary, just 5.4% of individuals reported high maladaptive coping and low adaptive coping and 3.3% reported high adaptive coping and low maladaptive coping.

**Table 3:** Prevalence of concurrent coping combinations among 426 PWH entering HIV care in Cameroon

Maladaptive coping tertile	Adaptive coping tertile		
	Low N (% of total sample)	Medium N (% of total sample)	High N (% of total sample)
Low	70 (16.4)	50 (11.7)	14 (3.3)
Medium	51 (12.0)	48 (11.3)	56 (13.1)
High	23 (5.4)	47 (11.0)	67 (15.7)

Across all mental health disorders assessed, greater maladaptive coping was associated with higher prevalence of mental health symptoms. The prevalence of moderate to severe depressive symptoms was 2.2%, 18.1%, and 40.9% among those in the lower, middle, and upper tertiles of maladaptive coping, respectively (Table 4). Similarly, the prevalence of moderate to severe anxiety symptoms was 6.0%, 19.5% and 33.1% and the prevalence of probable PTSD symptoms was 2.2%, 12.9%, and 32.1% among those in the lower, middle, and upper tertiles of maladaptive coping, respectively. In contrast, level of adaptive coping was not significantly associated with any of the mental health outcomes assessed.

**Table 4:** Reported symptoms of depression, anxiety, and post-traumatic stress disorder (PTSD) by adaptive and maladaptive coping strategy scores

	Total N (col %)	Depressive symptoms	Anxiety symptoms*	PTSD symptoms
		Yes N (row %)	Yes N (row %)	Yes N (row %)
Maladaptive coping				
Lower tertile	134 (31.5)	3 (2.2)	8 (6.0)	3 (2.2)
Middle tertile	155 (36.4)	28 (18.1)	30 (19.5)	20 (12.9)
Upper tertile	137 (32.2)	56 (40.9)	45 (33.1)	44 (32.1)
Adaptive coping				
Lower tertile	144 (33.8)	23 (16.0)	30 (21.0)	16 (11.1)
Middle tertile	145 (34.0)	36 (24.8)	30 (20.7)	27 (18.6)
Upper tertile	137 (32.2)	28 (20.4)	23 (16.9)	24 (17.5)

*Missing: anxiety n = 2

In multivariable analyses greater maladaptive coping was associated with significantly greater prevalence of all mental health outcomes assessed (Table 5). Individuals in the upper tertile of maladaptive coping had 2.7 (95% CI 1.8, 3.9) times the prevalence of moderate to severe depressive symptoms, 2.2 (95% CI 1.4, 3.3) times the prevalence of moderate to severe anxiety symptoms, and 3.4 (95% CI 2.0, 5.5) times the prevalence of probable PTSD symptoms compared to those in the lower two tertiles of maladaptive coping. Adaptive coping was not associated with symptoms of any of the mental health disorders assessed in bivariate or multivariable models.

**Table 5:** Unadjusted and adjusted associations between high adaptive and maladaptive coping and current symptoms of depression, anxiety, and post-traumatic stress disorder (PTSD) among 426 adults entering HIV care in Cameroon

	Depression		Anxiety^a^		PTSD	
	uPR (95% CI)	aPR (95% CI)^b^	uPR (95% CI)	aPR (95% CI)^b^	uPR (95% CI)	aPR (95% CI)^b^
Maladaptive coping						
Lower 2 tertiles	REF	REF	REF	REF	REF	REF
Upper tertile	3.81 (2.58, 5.62)*	2.67 (1.84, 3.89)*	2.51 (1.71, 3.67)*	2.17 (1.43, 3.29)*	4.04 (2.54, 6.40)*	3.37 (2.04, 5.54)*
Adaptive coping						
Lower 2 tertiles	REF	REF	REF	REF	REF	REF
Upper tertile	1.00 (0.67, 1.50)	0.92 (0.60, 1.42)	0.81 (0.53, 1.25)	0.91 (0.56, 1.48)	1.18 (0.75, 1.86)	1.55 (0.98, 2.47)

*uPR* unadjusted prevalence ratio; *aPR* adjusted prevalence ratio; *CI* confidence interval

^a^Missing: anxiety n = 2

^b^adjusted for clinic (fixed effect), age (categorical), gender

*Significant at p < 0.001

## Discussion

In this study, we explored coping strategies and the psychometric properties of the Brief COPE among PWH initiating HIV care in Cameroon. In the exploratory factor analysis, we found 22-items from the Brief Cope clustered around two key domains: ‘adaptive’ and ‘maladaptive’ coping. Greater maladaptive coping was associated with greater prevalence of symptoms of depression, anxiety, and PTSD. In contrast, adaptive coping was not significantly associated with any of the mental health outcomes assessed. 

In this revised version of the Brief COPE, two items each related to distraction and substance use were removed, along with one item each related to humor and acceptance. Interestingly, even though distraction was not included in the maladaptive factor, distraction was commonly endorsed as a coping strategy among this group of PWH in Cameroon with 46% reporting that they turned to work or other activities to take one’s mind off things and 65% reporting that they do something to think about stressful situations less. This is somewhat similar to research with PWH in Nigeria which found that 75% of participants reported using distraction to cope and research with pregnant women with HIV in South Africa which found that distraction was the most frequently used maladaptive coping strategy reported [55, 56]. On the contrary, substance use, the other coping strategy removed from our modified version of the Brief COPE, was the least commonly endorsed coping strategy with less than 15% of participants reporting using alcohol or other drugs as a coping strategy. More research is needed to understand the ways in which distraction and substance use function as coping mechanisms among PWH in Cameroon and the relationship among substance use, distraction, and other maladaptive coping strategies.

Religion was the most frequently used coping strategy among this group of PWH in Cameroon with 70% reporting using religion to cope. This is consistent with previous research that has found religion to be among the most commonly used strategies to deal with stress or mental health concerns among PWH in SSA. Research with women with HIV in South Africa and PWH in Ethiopia and Zimbabwe found that religion was among the most frequently reported coping strategies [34, 57, 58]. Similarly, research with women (with and without HIV) in Ethiopia found that 75% reported using religion to cope with depression [59]. Interestingly, prior research with this same group of PWH in Cameroon found that religious leaders were the most commonly reported source of mental health support with 40% of participants with symptoms of a mental health or substance use disorder reporting seeking mental health-related support from religious leaders [60]. In recognition of the important role of religious leaders and faith healers across global settings, the 2013–2030 World Health Organization Mental Health Action Plan recommended greater collaboration with religious leaders and faith healers as mental health treatment resources [61]. Religious leaders can play an important role in reducing mental health- and HIV-related stigma, promoting adaptive coping strategies, providing culturally appropriate, safe, and effective mental health treatment, and reducing the mental health treatment gap [62]. Greater collaboration between religious leaders and the medical community has the potential to increase access to mental health care, destigmatize mental health disorders and treatment, and enhance the cultural relevance of mental health treatment [62]. Research to advance understanding of acceptable and sustainable methods of collaboration between religious leaders and the medical community is needed, particularly in SSA.

In multivariable analyses, endorsing a high level of maladaptive coping was associated with significantly greater prevalence of depression, anxiety, and PTSD symptoms. However, endorsing a high level of adaptive coping strategies was not associated with the prevalence of symptoms of depression, anxiety, or PTSD. Similar to current study findings, previous research has found maladaptive coping strategies to be positively associated with mental health symptoms. For example, studies with PWH in Botswana and pregnant women with HIV in South Africa found that maladaptive coping strategies were positively associated with depressive symptoms [33, 34]. A study with PWH in Vietnam found that maladaptive adaptive coping strategies were associated with depression [63]. Contrary to current findings, prior research has found adaptive coping strategies to be associated with lower prevalence of mental health symptoms [22, 23]. Further research is needed to better understand the relationship between adaptive coping and mental health in this context.

Coping is a multidimensional process. Participants concurrently endorsed a variety of coping mechanisms, both adaptive and maladaptive. A US-based study of men who have sex with men (MSM) living with HIV similarly found that the concurrent use of adaptive and maladaptive coping strategies was commonly reported. Importantly, this US-based study also found that greater use of maladaptive coping strategies was associated with higher prevalence of depressive symptoms, regardless of use of adaptive coping strategies [64]. Our research aligns with these findings and suggests that in the presence of high maladaptive coping, concurrent adaptive coping may be insufficient to improve mental health.

Interventions that seek to reduce maladaptive coping strategies have the potential to improve the mental health of PWH in Cameroon and may be more effective than interventions focused exclusively on enhancing adaptive coping. However, few coping-focused interventions have been developed and implemented with PWH in SSA. A coping-focused intervention with women in South Africa with HIV and sexual abuse histories was associated with reduced PTSD symptoms and increased social or spiritual coping [65]. A coping-focused intervention for bereaved PWH in the U.S. was associated with decreased depression. Further, this relationship was mediated through avoidant coping [66]. That is, the intervention was associated with decreased avoidant coping which, in turn, was associated with decreased depression [66]. A coping intervention with PWH in the U.S. who experienced childhood sexual abuse was associated with reduced PTSD symptoms and alcohol use [67, 68]. Given the well-documented global mental health treatment gap and the modifiable nature of coping strategies, coping-focused interventions, particularly ones that can be task-shifted and delivered by non-specialized providers, present a promising and feasible strategy to improve the mental health of PWH in SSA and other resource-limited settings.

This study has limitations worth noting. Data were collected from three urban HIV treatment facilities in Cameroon and may not be fully representative of other areas of Cameroon or SSA. In addition, all participants were initiating HIV care at the time of study enrollment, and time of first HIV-positive diagnosis was unavailable for study participants. Coping strategies may differ based on time since diagnosis and time in HIV care. Finally, all data are cross-sectional. As such, the causal relationship between coping mechanisms and symptoms of mental health disorders cannot be assessed.

Our study found that PWH endorsed a range of concurrent adaptive and maladaptive coping strategies. Greater maladaptive coping was associated with worse mental health across all outcomes assessed. Future efforts should explore the extent to which coping strategies change throughout the HIV care continuum. Interventions to reduce maladaptive coping have the potential to improve the mental health of PWH in Cameroon.

## Data Availability

Data cannot be made publicly available due to participant privacy restrictions. Upon request, data are available to interested parties pending IRB approval from the University of North Carolina and the National Ethics Committee of Cameroon. Requests for the data can be sent to irb_questions@unc.edu.

## Code Availability

Code used in these analyses is available upon request from LF.

## References

[1] Marcus JL, Leyden WA, Alexeeff SE, Anderson AN, Hechter RC, Hu H (2020). Comparison of overall and comorbidity-free life expectancy between insured adults with and without HIV infection, 2000–2016. JAMA Netw Open.

[2] Smiley CL, Rebeiro PF, Cesar C, Belaunzaran-Zamudio PF, Crabtree-Ramirez B, Padgett D (2021). Estimated life expectancy gains with antiretroviral therapy among adults with HIV in Latin America and the Caribbean: a multisite retrospective cohort study. Lancet HIV.

[3] Kumar V, Singh J, Singh H (2021). A randomized prospective study to assess health-related quality-of-life outcomes of antiretroviral therapy in human immunodeficiency virus-positive adults. Indian J Sex Transm Dis AIDS.

[4] Andersson N, Cockcroft A, Shea B (2008). Gender-based violence and HIV: relevance for HIV prevention in hyperendemic countries of southern Africa. AIDS.

[5] Mbonu NC, van den Borne B, De Vries NK (2009). Stigma of people with HIV/AIDS in Sub-Saharan Africa: a literature review. J Trop Med.

[6] Hatcher AM, Weiser SD, Cohen CR, Hagey J, Weke E, Burger R (2021). Food insecurity and intimate partner violence among HIV-positive individuals in rural Kenya. Am J Prev Med.

[7] MacLean JR, Wetherall K (2021). The association between HIV-stigma and depressive symptoms among people living with HIV/AIDS: A systematic review of studies conducted in South Africa. J Affect Disord.

[8] Meskele M, Khuzwayo N, Taylor M (2021). Mapping the evidence of intimate partner violence among women living with HIV/AIDS in sub-Saharan Africa: a scoping review. BMJ Open.

[9] Nigusso FT, Mavhandu-Mudzusi AH (2021). High magnitude of food insecurity and malnutrition among people living with HIV/AIDS in Ethiopia: a call for integration of food and nutrition security with HIV treatment and care Programme. Nutr Health.

[10] Brandt R (2009). The mental health of people living with HIV/AIDS in Africa: a systematic review. Afr J AIDS Res.

[11] Remien RH, Stirratt MJ, Nguyen N, Robbins RN, Pala AN, Mellins CA (2019). Mental health and HIV/AIDS: the need for an integrated response. AIDS.

[12] Tuthill EL, Sheira LA, Palar K, Frongillo EA, Wilson TE, Adedimeji A (2019). Persistent food insecurity is associated with adverse mental health among women living with or at risk of HIV in the United States. J Nutrition.

[13] Tsai AC, Wolfe WR, Kumbakumba E, Kawuma A, Hunt PW, Martin JN (2016). Prospective study of the mental health consequences of sexual violence among women living With HIV in rural Uganda. J Interpers Violence.

[14] Katz IT, Ryu AE, Onuegbu AG, Psaros C, Weiser SD, Bangsberg DR (2013). Impact of HIV-related stigma on treatment adherence: systematic review and meta-synthesis. J Int AIDS Soc.

[15] Parcesepe AM, Filiatreau LM, Ebasone PV, Dzudie A, Ajeh R, Wainberg M (2021). Gender, mental health, and entry into care with advanced HIV among people living with HIV in Cameroon under a national 'Treat All' policy. AIDS Behav.

[16] Parcesepe AM, Filiatreau LM, Ebasone PV, Dzudie A, Pence BW, Wainberg M (2021). Mental health and initiation of antiretroviral treatment at enrolment into HIV care in Cameroon under a national "treat all" policy: a cross-sectional analysis. J Int AIDS Soc.

[17] Benzekri NA, Sambou JF, Ndong S, Diallo MB, Tamba IT, Faye D (2021). The impact of food insecurity on HIV outcomes in Senegal, West Africa: a prospective longitudinal study. BMC Public Health.

[18] Folkman S (1984). Personal control and stress and coping processes: a theoretical analysis. J Pers Soc Psychol.

[19] McCarty CA, Weisz JR, Wanitromanee K, Eastman KL, Suwanlert S, Chaiyasit W (1999). Culture, coping, and context: Primary and secondary control among Thai and American youth. J Child Psychol.

[20] De Vaus J, Hornsey MJ, Kuppens P, Bastian B (2018). Exploring the East-West divide in prevalence of affective disorder: a case for cultural differences in coping with negative emotion. Pers Social Psychol Rev.

[21] Zeidner M, Saklofske D (1996). Adaptive and maladaptive coping. Handbook of coping: theory, research, applications.

[22] Faulk KE, Gloria CT, Steinhardt MA (2013). Coping profiles characterize individual flourishing, languishing, and depression. Anxiety Stress Coping.

[23] Kato T (2015). The impact of coping flexibility on the risk of depressive symptoms. PLoS ONE.

[24] Camargo CC, Cavassan NRV, Tasca KI, Meneguin S, Miot HA, Souza LR (2019). Depression and coping are associated with failure of adherence to antiretroviral therapy among people living with HIV/AIDS. AIDS Res Hum Retroviruses.

[25] Lyimo RA, Stutterheim SE, Hospers HJ, de Glee T, van der Ven A, de Bruin M (2014). Stigma, disclosure, coping, and medication adherence among people living with HIV/AIDS in Northern Tanzania. AIDS Patient Care STDs.

[26] Shrestha S, Shibanuma A, Poudel KC, Nanishi K, Koyama Abe M, Shakya SK (2019). Perceived social support, coping, and stigma on the quality of life of people living with HIV in Nepal: a moderated mediation analysis. AIDS Care.

[27] Groth N, Schnyder N, Kaess M, Markovic A, Rietschel L, Moser S (2019). Coping as a mediator between locus of control, competence beliefs, and mental health: a systematic review and structural equation modelling meta-analysis. Behav Res Therapy.

[28] Wardell JD, Shuper PA, Rourke SB, Hendershot CS (2018). Stigma, coping, and alcohol use severity among People living With HIV: a Prospective Analysis of Bidirectional and Mediated Associations. Ann Behav Med.

[29] Earnshaw VA, Bogart LM, Laurenceau JP, Chan BT, Maughan-Brown BG, Dietrich JJ (2018). Internalized HIV stigma, ART initiation and HIV-1 RNA suppression in South Africa: exploring avoidant coping as a longitudinal mediator. J Int AIDS Soc.

[30] Norcini Pala A, Steca P (2015). Illness perceptions and coping strategies among individuals diagnosed with HIV. J Behav Med.

[31] Weaver KE, Llabre MM, Durán RE, Antoni MH, Ironson G, Penedo FJ (2005). A stress and coping model of medication adherence and viral load in HIV-positive men and women on highly active antiretroviral therapy (HAART). Health Psychol.

[32] Moskowitz JT, Hult JR, Bussolari C, Acree M (2009). What works in coping with HIV? A meta-analysis with implications for coping with serious illness. Psychol Bull.

[33] Vavani B, Kraaij V, Spinhoven P, Amone-P'Olak K, Garnefski N (2020). Intervention targets for people living with HIV and depressive symptoms in Botswana. Afr J AIDS Res.

[34] Kotzé M, Visser M, Makin J, Sikkema K, Forsyth B (2013). Psychosocial variables associated with coping of HIV-positive women diagnosed during pregnancy. AIDS Behav.

[35] Seffren V, Familiar I, Murray SM, Augustinavicius J, Boivin MJ, Nakasujja N (2018). Association between coping strategies, social support, and depression and anxiety symptoms among rural Ugandan women living with HIV/AIDS. AIDS Care.

[36] Parcesepe AM, Mugglin C, Nalugoda F, Bernard C, Yunihastuti E, Althoff K (2018). Screening and management of mental health and substance use disorders in HIV treatment settings in low- and middle-income countries within the global IeDEA consortium. J Int AIDS Soc.

[37] Parcesepe AM, Lancaster K, Edelman EJ, DeBoni R, Ross J, Atwoli L (2020). Substance use service availability in HIV treatment programs: Data from the global IeDEA consortium, 2014–2015 and 2017. PLoS ONE.

[38] Carver CS (1997). You want to measure coping but your protocol's too long: consider the brief COPE. Int J Behav Med.

[39] Kroenke K, Spitzer RL, Williams JB (2001). The PHQ-9: validity of a brief depression severity measure. J Gen Internal Med.

[40] Pence BW, Gaynes BN, Atashili J, O'Donnell JK, Tayong G, Kats D (2012). Validity of an interviewer-administered patient health questionnaire-9 to screen for depression in HIV-infected patients in Cameroon. J Affective Disorders.

[41] Monahan PO, Shacham E, Reece M, Kroenke K, Ong'or WO, Omollo O (2009). Validity/reliability of PHQ-9 and PHQ-2 depression scales among adults living with HIV/AIDS in western Kenya. J Gen Internal Med.

[42] Akena D, Joska J, Obuku EA, Stein DJ (2013). Sensitivity and specificity of clinician administered screening instruments in detecting depression among HIV-positive individuals in Uganda. AIDS Care.

[43] Carballeira Y, Dumont P, Borgacci S, Rentsch D, de Tonnac N, Archinard M (2007). Criterion validity of the French version of Patient Health Questionnaire (PHQ) in a hospital department of internal medicine. Psychol Psychotherapy.

[44] Spitzer RL, Kroenke K, Williams JB, Lowe B (2006). A brief measure for assessing generalized anxiety disorder: the GAD-7. Arch Internal Med.

[45] Chibanda D, Verhey R, Gibson LJ, Munetsi E, Machando D, Rusakaniko S (2016). Validation of screening tools for depression and anxiety disorders in a primary care population with high HIV prevalence in Zimbabwe. J Affective Dis.

[46] Zhong QY, Gelaye B, Zaslavsky AM, Fann JR, Rondon MB, Sánchez SE (2015). Diagnostic validity of the generalized anxiety disorder-7 (GAD-7) among Pregnant Women. PLoS ONE.

[47] Ruiz MA, Zamorano E, García-Campayo J, Pardo A, Freire O, Rejas J (2011). Validity of the GAD-7 scale as an outcome measure of disability in patients with generalized anxiety disorders in primary care. J Affective Dis.

[48] Micoulaud-Franchi JA, Lagarde S, Barkate G, Dufournet B, Besancon C, Trébuchon-Da Fonseca A (2016). Rapid detection of generalized anxiety disorder and major depression in epilepsy: validation of the GAD-7 as a complementary tool to the NDDI-E in a French sample. Epilepsy Behav.

[49] Blevins CA, Weathers FW, Davis MT, Witte TK, Domino JL (2015). The posttraumatic stress Disorder Checklist for DSM-5 (PCL-5): development and initial psychometric evaluation. J Traumatic Stress.

[50] Bovin MJ, Marx BP, Weathers FW, Gallagher MW, Rodriguez P, Schnurr PP (2016). Psychometric properties of the PTSD Checklist for Diagnostic and Statistical Manual of Mental Disorders-Fifth Edition (PCL-5) in veterans. Psychol Assess.

[51] Verhey R, Chibanda D, Gibson L, Brakarsh J, Seedat S (2018). Validation of the posttraumatic stress disorder checklist-5 (PCL-5) in a primary care population with high HIV prevalence in Zimbabwe. BMC Psychiatry.

[52] Ashbaugh AR, Houle-Johnson S, Herbert C, El-Hage W, Brunet A (2016). Psychometric validation of the English and French versions of the posttraumatic stress Disorder Checklist for DSM-5 (PCL-5). PLoS ONE.

[53] Shrive FM, Stuart H, Quan H, Ghali WA (2006). Dealing with missing data in a multi-question depression scale: a comparison of imputation methods. BMC Med Res Methodol.

[54] Child D. The Essentials of Factor Analysis. Third edition ed. London: Continuum International Publishing Group; 2006.

[55] Ahmed A, Osinubi MO, Fasiku MM, Uthman MM, Soyannwo T, Jimoh OS (2021). Coping strategies among patients attending HIV clinics in a North-central State of Nigeria. Nigerian J Clin Pract.

[56] Kotzé M, Visser M, Makin J, Sikkema K, Forsyth B (2013). The coping strategies used over a two-year period by HIV-positive women who had been diagnosed during pregnancy. AIDS Care.

[57] Ataro Z, Mengesha MM, Abrham A, Digaffe T (2020). Gender differences in perceived stigma and coping strategies among people living with HIV/AIDS at Jugal Hospital, Harar, Ethiopia. Psychol Res Behav Manag.

[58] Tarwireyi F (2005). Stigma and discrimination: coping behaviours of people living with HIV and AIDS in an urban community of Mabvuku and Tafara, Harare, Zimbabwe. Central Afr J Med.

[59] Azale T, Fekadu A, Medhin G, Hanlon C (2018). Coping strategies of women with postpartum depression symptoms in rural Ethiopia: a cross-sectional community study. BMC Psychiatry.

[60] Filiatreau LM, Ebasone PV, Dzudie A, Ajeh R, Pence B, Wainberg M (2021). Correlates of self-reported history of mental health help-seeking: a cross-sectional study among individuals with symptoms of a mental or substance use disorder initiating care for HIV in Cameroon. BMC Psychiatry.

[61] World Health Organization (2021). Comprehensive mental health action plan, 2013–2030.

[62] Pham TV, Koirala R, Wainberg ML, Kohrt BA (2021). Reassessing the mental health treatment gap: what happens if we include the impact of traditional healing on mental illness?. Commun Mental Health J.

[63] Matsumoto S, Yamaoka K, Nguyen HDT, Nguyen DT, Nagai M, Tanuma J (2020). Validation of the Brief Coping Orientation to Problem Experienced (Brief COPE) inventory in people living with HIV/AIDS in Vietnam. Global Health Med.

[64] Rood BA, McConnell EA, Pantalone DW (2015). Distinct coping combinations are associated with depression and support service utilization in men who have sex with men living with HIV. Psychol Sex Orientat Gend Divers.

[65] Sikkema KJ, Mulawa MI, Robertson C, Watt MH, Ciya N, Stein DJ (2018). Improving AIDS care after trauma (ImpACT): pilot outcomes of a coping intervention among HIV-infected women with sexual trauma in South Africa. AIDS Behav.

[66] Smith NG, Tarakeshwar N, Hansen NB, Kochman A, Sikkema KJ (2009). Coping mediates outcome following a randomized group intervention for HIV-positive bereaved individuals. J Clinical Psychol.

[67] Sikkema KJ, Hansen NB, Kochman A, Tarakeshwar N, Neufeld S, Meade CS (2007). Outcomes from a group intervention for coping with HIV/AIDS and childhood sexual abuse: reductions in traumatic stress. AIDS Behav.

[68] Meade CS, Drabkin AS, Hansen NB, Wilson PA, Kochman A, Sikkema KJ (2010). Reductions in alcohol and cocaine use following a group coping intervention for HIV-positive adults with childhood sexual abuse histories. Addiction.

